# Viroids, Satellite RNAs and Prions: Folding of Nucleic Acids and Misfolding of Proteins

**DOI:** 10.3390/v16030360

**Published:** 2024-02-26

**Authors:** Gerhard Steger, Detlev Riesner, Stanley B. Prusiner

**Affiliations:** 1 Institut für Physikalische Biologie, Faculty of Mathematics and Natural Sciences, Heinrich Heine University Düsseldorf, 40204 Düsseldorf, Germany; detlev.riesner@hhu.de; 2 Institute for Neurodegenerative Diseases, Weill Institute for Neurosciences, University of California San Francisco, San Francisco, CA 94158, USA; stanley.prusiner@ucsf.edu; 3 Department of Neurology, Weill Institute for Neurosciences, University of California San Francisco, San Francisco, CA 94158, USA; 4 Department of Biochemistry and Biophysics, University of California San Francisco, San Francisco, CA 94158, USA

**Keywords:** potato spindle tuber viroid, *Pospiviroidae*, *Avsunviroidae*, virusoid, satellite RNA of cucumber mosaic virus, prion diseases

## Abstract

Theodor (“Ted”) Otto Diener (* 28 February 1921 in Zürich, Switzerland; † 28 March 2023 in Beltsville, MD, USA) pioneered research on viroids while working at the Plant Virology Laboratory, Agricultural Research Service, USDA, in Beltsville. He coined the name viroid and defined viroids’ important features like the infectivity of naked single-stranded RNA without protein-coding capacity. During scientific meetings in the 1970s and 1980s, viroids were often discussed at conferences together with other “subviral pathogens”. This term includes what are now called satellite RNAs and prions. Satellite RNAs depend on a helper virus and have linear or, in the case of virusoids, circular RNA genomes. Prions, proteinaceous infectious particles, are the agents of scrapie, kuru and some other diseases. Many satellite RNAs, like viroids, are non-coding and exert their function by thermodynamically or kinetically controlled folding, while prions are solely host-encoded proteins that cause disease by misfolding, aggregation and transmission of their conformations into infectious prion isoforms. In this memorial, we will recall the work of Ted Diener on subviral pathogens.

## 1. Introduction

**Figure 1:**
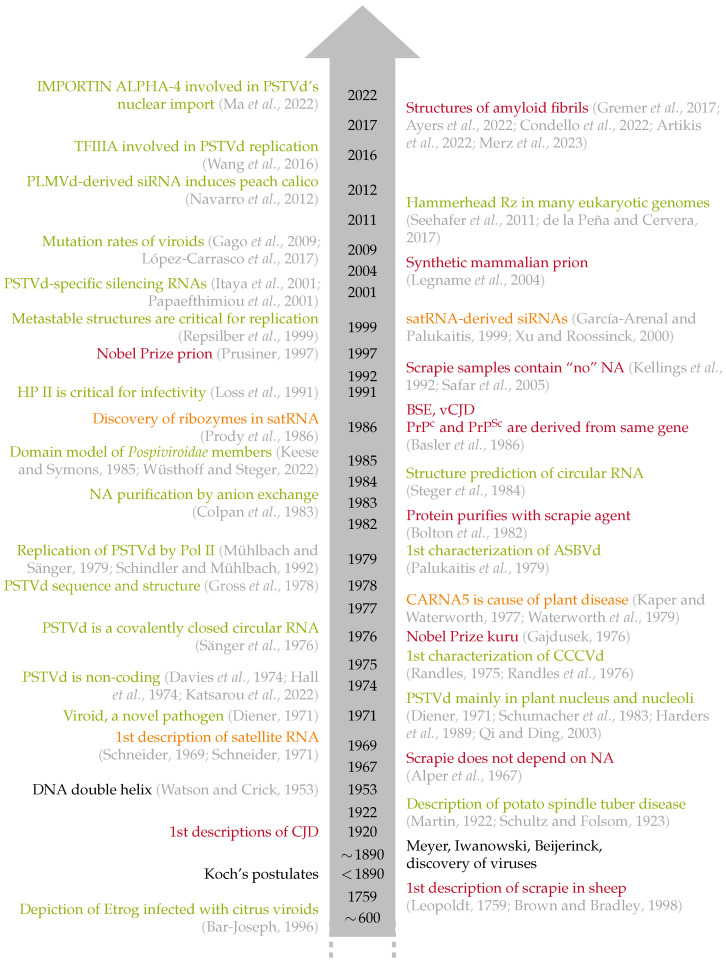
Timeline of research development of viroids (green text) [4,13,14,15,16,17,18,19,20,21,22,23,24,25,26,27,28,29,30,31,32,33,34,35,36,37,38,39,40,41,42,43,44], satellite RNAs (orange text) [11,12,45,46,47,48,49], prions (red text) [7,50,51,52,53,54,55,56,57,58,59,60,61,62,63], and a few general hallmarks (black text) [64].

‘Dogma’ is generally defined as a principle or set of principles thought to be true. A well-known dogma formulated by Francis Crick in 1958 [1,2] stated that biological information flows from DNA to RNA to proteins. With the discovery of reverse transcription, the first exception became known. Another example of an outdated dogma was that viruses—and phages—were the smallest infectious agents. They possess a nucleic acid genome that encodes a few proteins. It was Theodor (“Ted”) O. Diener who first described a naked RNA, which is 5 to 10-fold smaller than the smallest known viral genome and has no coding capacity [3,4,5,6]. More unexpected was the discovery of proteinaceous particles called prions, which were first discovered in studies of the infectious agent that causes scrapie of sheep [7,8]. Notably, Diener was extremely skeptical about prions initially [9], but he later collaborated with Prusiner on a study that compared the properties of viroids and prions [10]. Of note, satellite RNAs are similar in size, structure, and pathological effects, but they do not autonomously replicate; rather, they need a specific virus, or helper virus, to multiply. Satellite RNAs were discovered at about the same time as viroids [11,12], and discussing their features here helps to clarify the differences among viroids, subviral satellites and prions. Figure 1 shows a timeline of discoveries focused on this article’s topics.

## 2. Viroids

In the late 1960s, Diener and co-workers described a free RNA that is infectious in plants, which causes potato spindle tuber disease [3,65]. They concluded that the RNA is too small to contain the genetic information necessary for self-replication and that it must rely on host enzymes for its replication. Joseph S. Semancik and Lewis G. Weathers reported similar findings for the causal agent of citrus exocortis disease [66,67,68,69]. For such an unconventional agent, Diener suggested the term viroid [4], which was adopted in 1972. In the 1970s, the purification methods for small RNAs improved substantially, in particular by ultracentrifugation and gel electrophoresis, and later by chromatography [25]. In Germany, Heinz Ludwig Sänger successfully isolated viroids from several viroid diseases in larger quantities [70,71,72]. He convinced the groups of Albrecht K. Kleinschmidt and Günther Klotz, Hans J. Gross, and Detlev Riesner to jointly study viroids. This cooperation of the “German team” [73] resulted in several breakthrough publications demonstrating the following properties: viroid RNA circularity, the molecular weight, secondary structure, and mechanism of structure formation [30,74]. Hans Gross and his group published the complete nucleotide sequence of the potato spindle tuber viroid (PSTVd) and confirmed its rod-shaped secondary structure in 1978 [29,75]. The biophysical studies demonstrated that certain extra-stable structural elements of viroids, named hairpins I and II, are not part of the native, thermodynamically favored structure; rather, they are present as metastable elements in kinetically favored structures during replication and are critical for replication and processing [22,23,76,77,78,79]. Furthermore, the structural studies contributed to easy, fast diagnostic methods [80] and were even used later to find or exclude nucleic acids in prions (see Section 4).

After the structural research, interest turned to the mechanism of viroid replication and pathogenicity. Ted Diener set the pace, and viroid research groups grew all over the world, including those who studied avocado sun blotch viroid (ASBVd) [28], coconut cadang-cadang viroid (CCCVd) [31,32], and others.

Today, viroids are classified into the families *Pospiviroidae* (named after PSTVd; 39 members) and *Avsunviroidae* (named after ASBVd; 5 members) “on the basis of their biological, biochemical and structural features. Members of the family *Avsunviroidae* can form, in the strands of both polarities, hammerhead ribozymes that mediate replication in chloroplasts, in which these viroids accumulate. Members of the family *Pospiviroidae* lack hammerhead ribozymes but contain a central conserved region (CCR) in their rod-like or quasi rod-like conformation and replicate in the nucleus, wherein these viroids also accumulate” [81].

## 3. Viroids: The Archetype of Further Subviral Pathogens?

**Figure 2:**
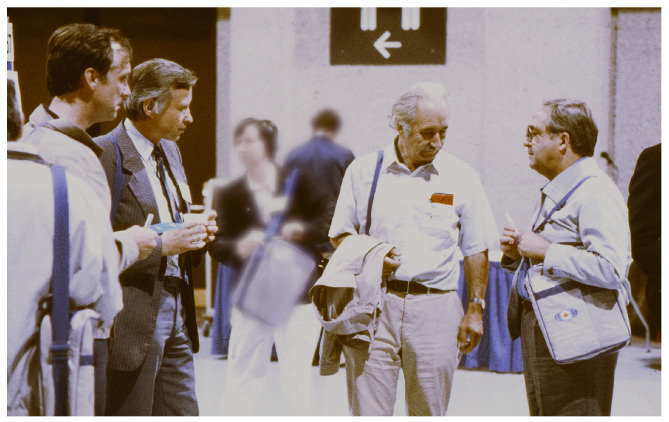
At the 7th International Conference of Virology in Edmonton, Canada, 1987. From left to right: Robert A. Owens, Detlev Riesner, Theodor O. Diener, Heinz Ludwig Sänger.

After the sequence of PSTVd and some early results about the replication mechanism were determined [26,27,82,83], viroids were established and accepted as subviral pathogens. The viroid researchers became a worldwide family essentially, meeting in Beltsville [84] and during many symposia (Figure 2). But during those symposia, other unknown pathogens were discussed including the agents of kuru [60,85], called unconventional virus, and scrapie, a sheep disease leading to amyloidosis. Similar to viroids, these particles could not be seen under a light microscope and nobody knew what to look for using an electron microscope since a molecular test did not exist. Could this type of unidentified infectious animal agents be a new class of viroids? Semancik, Hanson and coworkers published two papers in *Nature* arguing that scrapie had an essential DNA component of small molecular weight [86,87]. Even Ted Diener similarly speculated in a contribution to the 2nd International Congress for Virology, Budapest (1971) [9]. After the groundbreaking publication of Stanley B. Prusiner in *Science* 1982, in which he coined the term “prion” [88], controversies were plentiful and discussed during joint sessions on subviral pathogens; the last one took place at the 6th International Congress for Virology in Sendai (1984). After that, the scientific directions split, but the earlier often controversial discussions helped clarify the fundamental differences between the plant and animal subviral pathogens.

For young scientists, the research on subviral pathogens remained very attractive, and Ted Diener and Detlev Riesner organized a summer school of the Studienstiftung des Deutschen Volkes (German Academic Scholarship Foundation) in Bled, Slovenia, on “Subviral pathogens: Viroids and Prions” in 1994.

## 4. Prions

**Figure 3:**
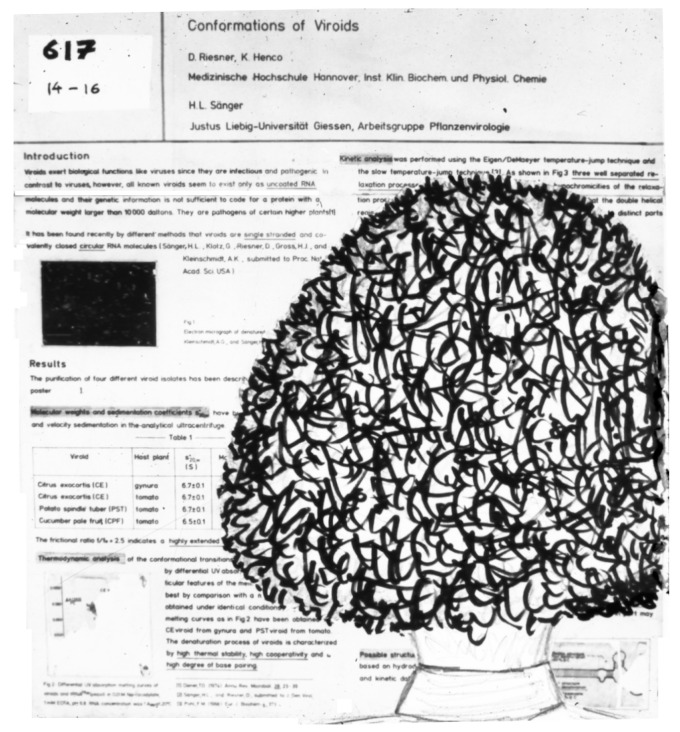
A poster and its visitor at the 10th International Congress of Biochemistry, Hamburg, Germany (1976). Prusiner in front of the poster sketched from memory by Riesner.

Stanley B. Prusiner systematically continued the earlier studies of Alper et al. [61] into the inactivation properties of scrapie infectivity; Latarjet et al. [89] had shown that the scrapie UV spectrum of inactivation is similar to that of proteins rather than that of nucleic acids. Several years later, Stanley Prusiner and Detlev Riesner had a prolonged discussion about viroids and prions prompted by Riesner’s “Viroid Poster” at the 10th International Congress of Biochemistry, Hamburg (1976). Prusiner came to study the poster and blocked the whole poster with his voluminous hairstyle (Figure 3). While nobody else could look at the poster, Prusiner and Riesner fell into an intensive discussion on viroids and the scrapie agent.

In 1978, during a meeting in Munich, Germany, organized by Sänger, Prusiner reported that the sedimentation behavior of scrapie infectivity was heterogeneous and different from viruses and viroids. Prusiner summarized the results of his subsequent studies: chemical and physical procedures that destroy nucleic acids do not destroy scrapie infectivity, while chemical and physical procedures that destroy proteins do destroy scrapie infectivity. In his groundbreaking publication in 1982 [88], he concluded: “Novel proteinaceous infectious particles cause scrapie”. Whereas viroids were already accepted, prions remained surrounded by considerable skepticism for many more decades. In contrast to most of the skeptics, Ted Diener, who had published a paper entitled “Is the scrapie agent a viroid” [9] in 1972, accepted the new data underpinning the prion hypothesis and published a paper on the fundamental differences between viroids and prions with Prusiner and his colleagues in 1982 [10].

Using some viroid research approaches, Riesner and colleagues applied the quantitative methods of nucleic acid analysis—even counting single molecules—and showed in systematic and extended studies that highly infectious samples from hamster brains contained more infectious units than nucleic acid molecules longer than 25 nucleotides [57,58]. We will not outline the whole research development on prions here; Prusiner was awarded the Nobel Prize “for his discovery of Prions—a new biological principle of infection” [56] in 1997, and the final molecular proof was found in 2004 when a synthetic prion—i. e., a synthetic protein that never has seen an animal—was created [55,90].

Due to the ongoing research of Prusiner’s group and others, the present day mechanism of prion amplification was determined. The prion protein (PrP) can exist in a cellular, non-pathological conformation (PrP^c^), which is mainly α-helical, or in an aggregated, pathological conformation (PrP^Sc^, i. e., scrapie), which has a higher proportion of β-sheets. PrP^c^ is expressed from a single-copy gene of the host [59] and presented on the outer surface of the cell. If PrP^c^ encounters invading PrP^Sc^ during infection, PrP^Sc^ forces PrP^c^ to misfold into the pathological conformation of PrP^Sc^. When PrP^Sc^ aggregates, then breaks apart and encounters nascent PrP^c^, a PrP^Sc^ dimer or oligomer is formed. This replication process continues until the accumulation of PrP^Sc^ prions kills the host cells.

The infectivity of prions results from a conformational change of PrP^c^ into PrP^Sc^. The first PrP^Sc^ particles can be generated spontaneously by the rare aggregation of several PrP^c^ molecules or can be facilitated by specific mutations. The foregoing process produces prion diseases that can originate spontaneously, by infection or as familial diseases. Prion disease was the first example in medicine of three origins of the same disease [91].

## 5. Satellites and Virusoids

At the conference in Munich in 1978, Jacobus (“Jap”) M. Kaper presented data that CARNA 5 (cucumber mosaic virus |CMV] associated RNA 5), the satellite RNA of CMV (satCMV), was able to modify CMV symptoms depending on CMV strain, CARNA 5 variant, and host plant [49,92]. Virions of CMV contain the three genomic RNAs 1–3, a subgenomic RNA 4, and sometimes a fifth RNA, giving rise to the name CARNA 5. Thus, an RNA was identified that is similar in size to viroids, can also induce pathological effects, and—in contrast to viroids—is linear, depending on a helper CMV to replicate and accumulating in planta in double-stranded form [93].

Plant infections with CMV and necrogenic CARNA 5 variants lead to systemic necrosis and destruction of plants [46,48], exemplified by several epidemics [45]. In contrast, infections with CMV and non-necrogenic variants show only marginal symptoms and a yield that is similar to that of non-infected plants, even above that of plants infected with CMV without a satellite. This opened the possibility to (cross-)protect crops by pre-infection or “vaccination” with CMV and non-necrogenic satellites [94,95]. It was important, however, to verify that during this biological control process, no necrogenic CARNA 5 variants were emerging; that is, the non-necrogenic variant should differ from any necrogenic variant by more than a single mutation [96]. Following this line, Tien Po (Academia Sinica, Beijing, China) analyzed double-stranded CARNA 5 (dsCARNA 5) variants during his sabbatical stay in Düsseldorf in 1986 and 1987. The major method for analysis was temperature-gradient gel electrophoresis (TGGE) [97,98] that can separate macromolecules of identical length that differ by a few or even single mutations, which influence their denaturation behavior. Indeed, several dsCARNA 5 samples consisted of different molecular species that were only separated at temperatures leading to a partial denaturation but co-migrated as dsRNA at low temperature and as single strands after full denaturation. Common to all samples were three transitions: two low-temperature transitions, due to partial denaturation leading to strong gel retardation, and a high-temperature transition into single strands, which migrated much faster than the partially denatured molecules. For the non-necrogenic variants, the transition at the lowest temperature was below the corresponding transition of the necrogenic variants [99,100].

Virusoids are small, circular, single-stranded satellite RNAs that are encapsidated by respective plant viruses and replicated by the viral polymerase; their native secondary structure is mostly rod-like with a few small bifurcations. John W. Randles, recipient of a Ludwig Leichhardt fellowship from the Alexander von Humboldt Foundation, arrived for his sabbatical in Düsseldorf with viroid and virusoid samples in 1981. Together, Randles, Steger and Riesner showed that CCCVd possesses thermodynamic and kinetic features of a typical viroid including an extra-stable hairpin I but not an extra-stable hairpin II. In contrast, the virusoids of subterranean clover mottle virus (satSNMV) and velvet tobacco mottle virus (satVTMoV) are thermodynamically less stable than viroids, despite similar GC content, and do not possess extra-stable hairpin(s) [24,101]. Thomas C. Goodman, a postdoctoral fellow of the Alexander von Humboldt Foundation, showed that DNA-dependent RNA polymerase II, the enzyme that replicates members of *Pospiviroidae*, binds PSTVd specifically at one end of its secondary structure. In contrast, satSNMV and satVTMoV are only nonspecifically bound, similar to tRNA, for example [102].

## 6. Outlook

The discovery of viroids was a strenuous dive into the new world of subviral pathogens and included a prolonged battle against the disbelief of many scientists. Consequently, we cannot forget the pioneering work of Theodor O. Diener and his successors: Joseph S. Semancik [69] and Heinz L. Sänger [70], who described first the citrus exocortis viroid; Rudra P. Singh and his work on PSTVd [103]; John W. Randles and his discovery of CCCVd [31,32]; and Robert H. Symons, who first described the ASBVd and its self-splicing by hammerhead ribozymes [28,104]. The discovery of new viroid(-like) RNAs has increased in recent years mostly due to the use of high-throughput sequencing [105,106,107,108].

Of course, the knowledge on these subviral particles has expanded greatly over the following half century. Viroids are now subdivided into the families *Pospiviroidae* and *Avsunviroidae*, which are located mainly in the nucleus and the chloroplast, respectively. Their locations also suggest their usage of different replication strategies, host polymerases, structural elements, and trafficking [13,109,110,111]. The knowledge on viroid-induced symptoms is increasing, but still not solved fully. At least in the case of peach latent mosaic viroid variants that induce an extensive chlorosis of peach, a specific viroid-derived small RNA is involved [15,112]; mechanisms are less clear in other cases [113,114]. Elimination of some viroids during pollen maturation involves a depression of viroid replication and an increase in degradation processes [115,116].

Current knowledge on replication of virusoids and satellite RNAs by the polymerase of their helper virus is discussed in recent reviews [117,118] showing that complex secondary and tertiary structures of satellite RNAs are involved in suppression of the helper virus and symptoms [119,120,121,122,123].

What started with research in plants and animals had later impact in general molecular biology, extending to human disease and therapeutic developments. Here follows a few spot lights:

- Chromatography for viroid purification [25] led to the development of plasmid purification kits [124,125,126], which are now used worldwide in molecular biology research.
- Hammerhead ribozymes have been detected in most genomes [16,127]. Retrotransposons with hammerhead ribozymes, called retrozymes, have been found encoded in diverse plant genomes [17] and have stimulated new ideas about the possible origin of viroid and viroid-like RNAs [128,129,130,131]. Viroid-like RNAs, termed mycoviroids, have been detected in fungi [132,133,134].
- Knowledge of circular RNAs in mammals has expanded in recent years; for example, they are produced by a process called back-splicing from mRNAs and are involved in (mis)regulation of many processes [135,136,137].
- Current knowledge on virusoids and other satellite RNAs has expanded substantially [117,118]. The human hepatitis delta virus satellite (HDV) [138,139] of hepatitis B virus (HBV) increases the fatality of hepatitis and is today the object of intensive therapeutic development [140,141].
- In the early 1980s, there was only a minor interest in rare neurodegenerative diseases like kuru, Creutzfeldt–Jakob and Gerstmann–Sträussler–Scheinker in humans and scrapie in sheep, which seems not to be transmissible to humans. This situation changed drastically with Prusiner’s concept of prions and the discovery of the bovine spongiform encephalopathy (BSE) epidemic in the mid-1980s. Later, variant Creutzfeldt–Jakob disease (vCJD) was found to be due to bovine ${\mathrm{PrP}}^{\mathrm{vCJD}}$ prions. With widespread testing of slaughtered cattle in Europe and elimination of bovine offal as a source of feed for cattle, sheep, and pigs, BSE has been eliminated from the roster of lethal human illnesses.

Much more than a spotlight is our knowledge of prions that is leading to effective therapeutics for Alzheimer’s and Parkinson’s diseases as well as related disorders. Recent breakthroughs in cryo-electron microscopy and solid-state NMR spectroscopy are helping elucidate the fibril structures of proteins that cause prion diseases [50,51,52,54,142]. Hopefully, by understanding the biophysics, molecular biology and protein interactions involved in these diseases, effective therapeutics can be developed.
